# Supplementary material for: The Antimicrobial Peptide AMP-IBP5 Suppresses Dermatitis-like Lesions in a Mouse Model of Atopic Dermatitis through the Low-Density Lipoprotein Receptor-Related Protein-1 Receptor
Source: Int J Mol Sci. 2023 Mar 8;24(6):5200. doi: 10.3390/ijms24065200 (PMC10049508; doi:10.3390/ijms24065200)
Supplement: Supplementary file 1 [file ijms-24-05200-s001.zip › ijms-2204378-supplementary.pdf]

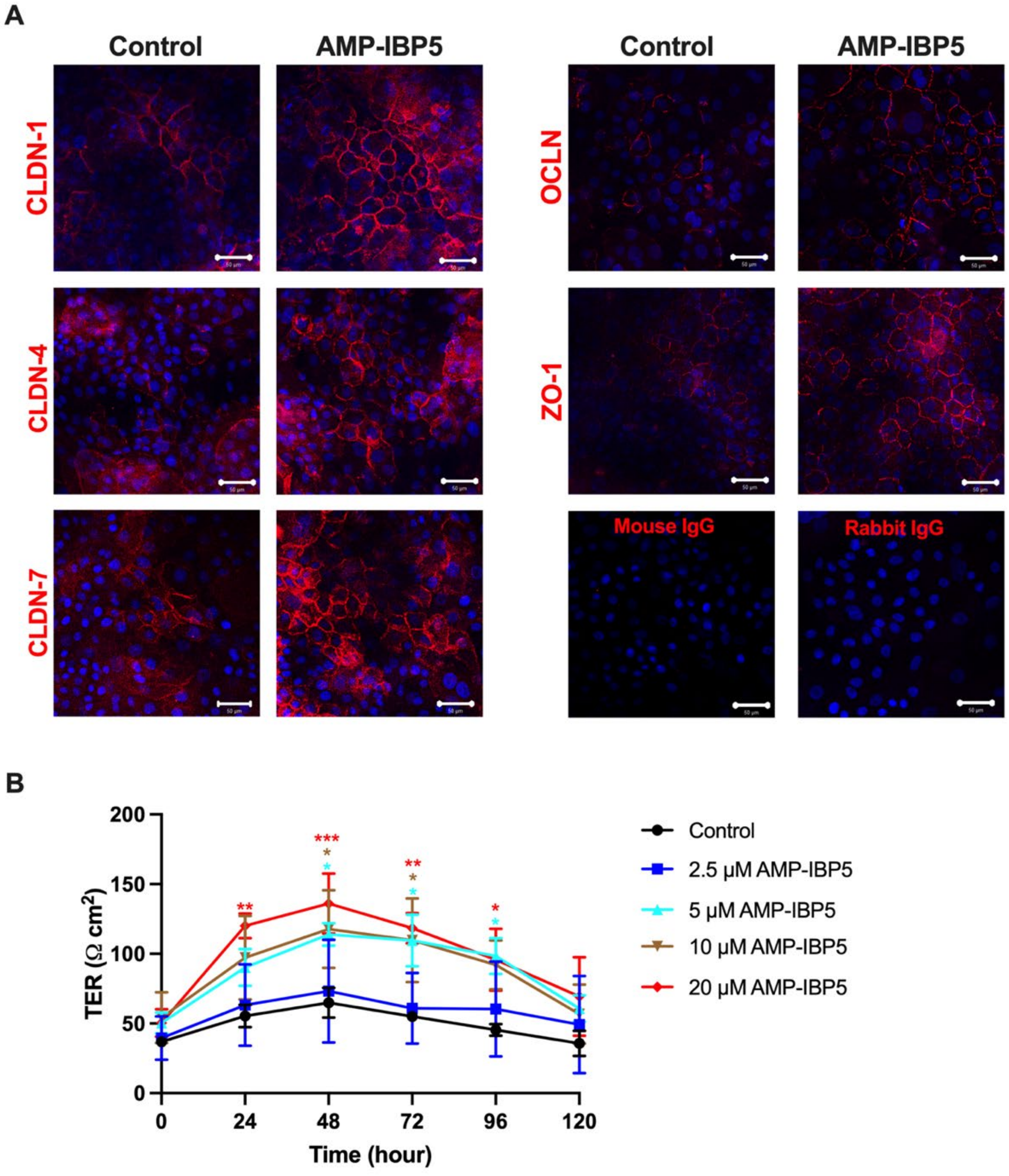


**Figure S1. AMP-IBP5 enhanced TJ barrier function in human keratinocytes.** (A) Human primary keratinocytes grown to confluence on collagen I-coated chamber slides were incubated with 10 μM AMP-IBP5 for 48 hours, and immunofluorescence staining of TJ-related proteins, including claudin-1, -4 and -7, occludin, ZO-1 and isotype control, upon stimulation with AMP-IBP5 is shown. Scale bar: 50 μm. (B) Keratinocyte layers grown on Transwell inserts were incubated with 2.5, 5, 10, or 20 μM AMP-IBP5 for 120 hours, and TER was measured by CellZscope. The data are presented as the means ± SDs. *$P$ < 0.05, **$P$ < 0.01, ***$P$ < 0.001. Statistical significance was determined by one-way ANOVA with Tukey's multiple comparisons test. All data are representative of three independent experiments.

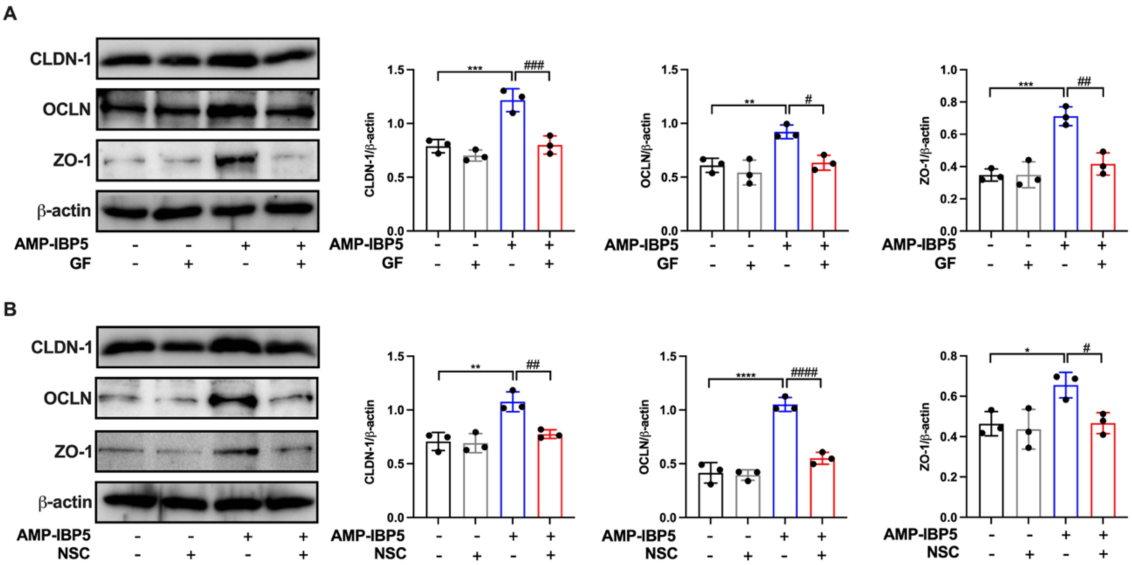


**Figure S2. The expression of TJ-related proteins was blocked by treatment with inhibitors of PKCζ and Rac1.** Human primary keratinocytes were pretreated with 100 nM GF 109203X (A) and 20 μM NSC23766 (B) for 24 hours, stimulated with 10 μM AMP-IBP5 for 48 hours and subjected to Western blotting using antibodies specific for claudin-1, occludin and ZO-1. Bands were quantified using densitometry; n=3/group. The data are presented as the means ± SDs. $^{*}P < 0.05$, $^{**}P < 0.01$, $^{***}P < 0.001$, $^{****}P < 0.0001$, $^{\#}P < 0.05$, $^{\#\#}P < 0.01$, $^{\#\#\#}P < 0.001$, $^{\#\#\#\#}P < 0.0001$. Statistical significance was determined by one-way ANOVA with Tukey's multiple comparisons test. All data are representative of three independent experiments.

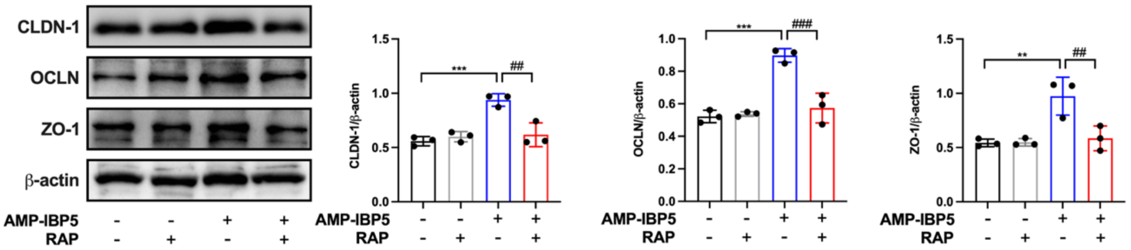


**Figure S3. The expression of TJ-related proteins was blocked by treatment with an LRP1 antagonist**. Human primary keratinocytes were pretreated with 1 μg/ml RAP for 24 hours, stimulated with 10 μM AMP-IBP5 for 48 hours and subjected to Western blotting using antibodies specific for claudin-1, occludin and ZO-1. Bands were quantified using densitometry; n=3/group. The data are presented as the means ± SDs. $^{**}P < 0.01$, $^{***}P < 0.001$, $^{\#\#}P < 0.01$, $^{\#\#\#}P < 0.001$. Statistical significance was determined by one-way ANOVA with Tukey's multiple comparisons test. All data are representative of three independent experiments.

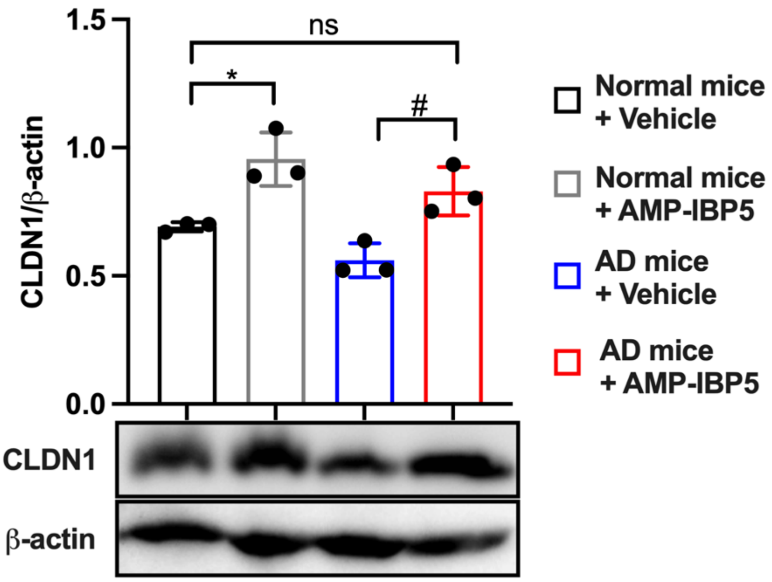


**Figure S4. Administration of AMP-IBP5 restored the expression of claudin-1 in AD mice.** The ear skin of the AD mice was subcutaneously injected with 25 µl of 25 µM AMP-IBP5 on Days 15, 17, and 18. On Day 19, the skin biopsies were harvested and subjected to Western blotting using antibodies specific for claudin-1. Bands were quantified using densitometry; n=3 mice/group. The data are presented as the means ± SDs. $^{*}P < 0.05$, $^{\#}P < 0.05$. Statistical significance was determined by one-way ANOVA with Tukey's multiple comparisons test. All data are representative of three independent experiments.

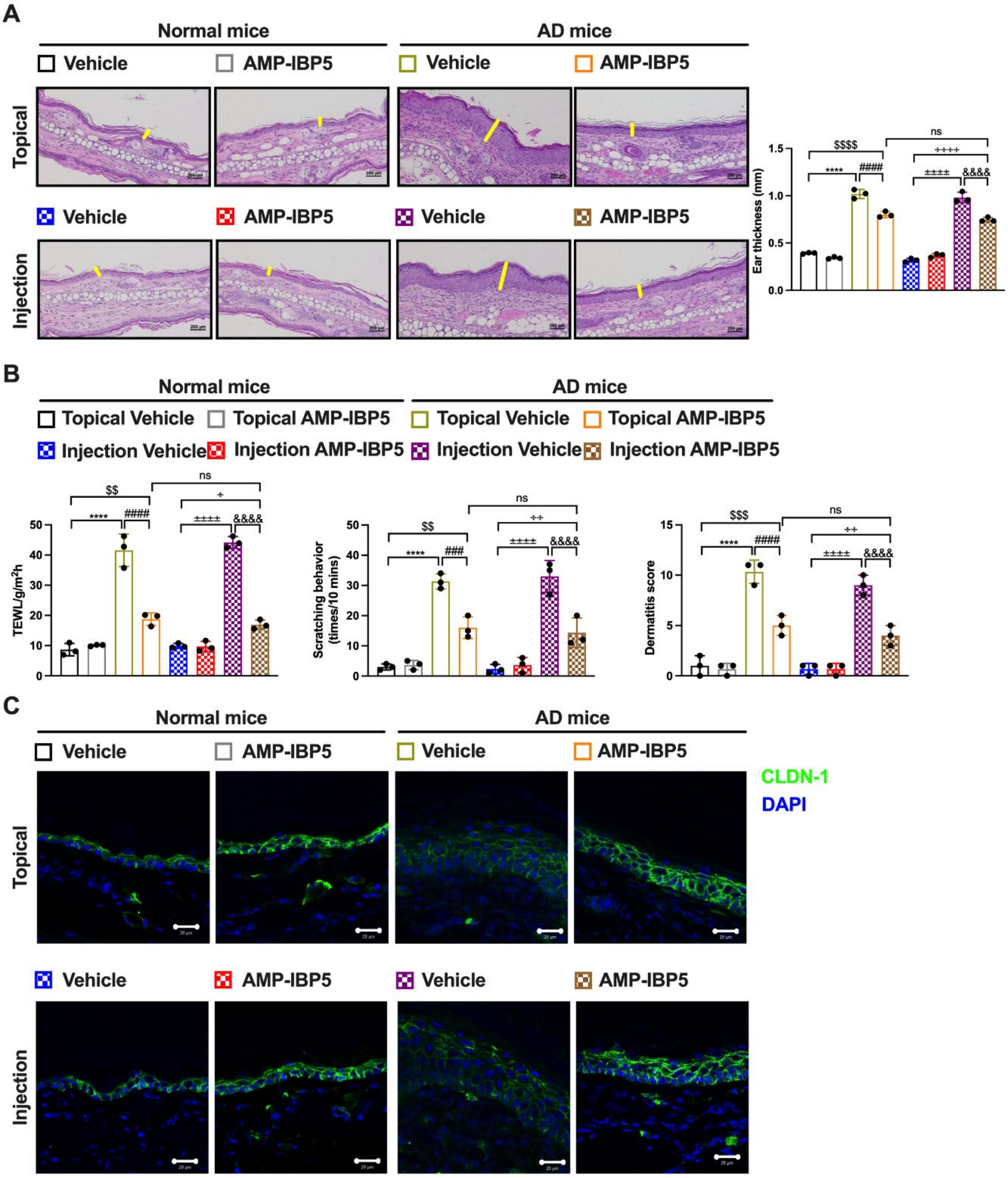


**Figure S5.** Topical application and injection of AMP-IBP5 alleviated dermatitis-like symptoms in AD mice to similar degrees. (A) Representative histological sections of mouse ears stained with H&E. The yellow lines indicate the epidermis (left panel, scale bars: 200 μm). Ears thickness of mice are shown (right panel, n=3 mice/group). (B) The dermatitis score, scratching behavior and transepidermal water loss (TEWL) of mice are shown. (C) Representative immunofluorescence images of claudin-1 in the mouse epidermis. Scale bar: 20 μm; n=3/group. $^{****}P < 0.0001$, $^{\#\#\#}P < 0.001$,$^{\#\#\#\#}P < 0.0001$, $^{\$\$}P < 0.01$, $^{\$\$\$}P < 0.001$, $^{\$\$\$\$}P < 0.0001$, $^{\pm\pm\pm\pm}P < 0.0001$, $^{+}P < 0.05$, $^{++}P < 0.01$, $^{++++}P < 0.0001$. Statistical significance was determined by one-way ANOVA with Tukey's multiple comparisons test. All data are representative of three independent experiments.

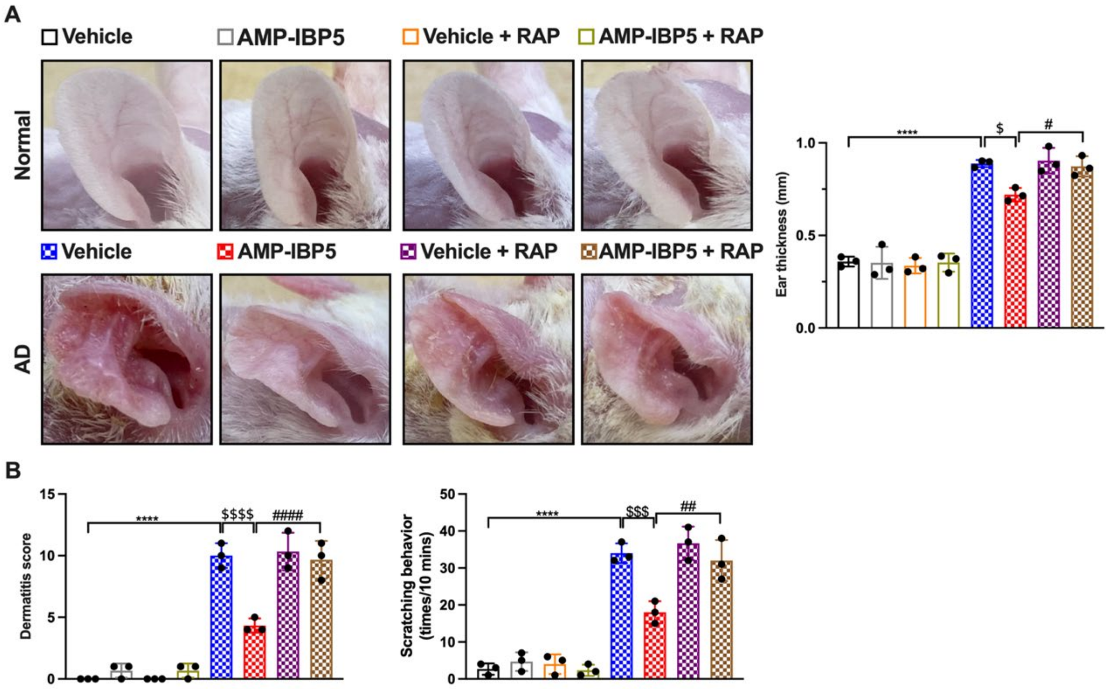


**Figure S6. The restorative effect of AMP-IBP5 on skin barrier function in mice is blocked by RAP injection.** (A) Representative images of the ears from mice (left) and the ear thickness (right) are depicted; n=3 mice/group. (B) The dermatitis scores and scratching behavior of AD mice are shown. The data are presented as the means ± SDs. $^{****}P < 0.0001$, $^{\#}P < 0.05$, $^{\#\#}P < 0.01$, $^{\#\#\#\#}P < 0.0001$, $^{\$}P < 0.05$, $^{\$\$\$}P < 0.001$, $^{\$\$\$\$}P < 0.0001$. Statistical significance was determined by one-way ANOVA with Tukey’s multiple comparisons test.
